# Effect of captivity on morphology: negligible changes in external morphology mask significant changes in internal morphology

**DOI:** 10.1098/rsos.172470

**Published:** 2018-05-09

**Authors:** Stephanie K. Courtney Jones, Adam J. Munn, Phillip G. Byrne

**Affiliations:** 1 Centre for Sustainable Ecosystem Solutions, School of Biological Sciences, University of Wollongong, Wollongong, New South Wales 2522, Australia; 2 School of Biological, Earth and Environmental Sciences, The University of New South Wales, Sydney New South Wales 2052, Australia

**Keywords:** captive breeding, morphology, reintroduction, phenotypic plasticity, conservation biology, captivity

## Abstract

Captive breeding programmes are increasingly relied upon for threatened species management. Changes in morphology can occur in captivity, often with unknown consequences for reintroductions. Few studies have examined the morphological changes that occur in captive animals compared with wild animals. Further, the effect of multiple generations being maintained in captivity, and the potential effects of captivity on sexual dimorphism remain poorly understood. We compared external and internal morphology of captive and wild animals using house mouse (*Mus musculus*) as a model species. In addition, we looked at morphology across two captive generations, and compared morphology between sexes. We found no statistically significant differences in external morphology, but after one generation in captivity there was evidence for a shift in the internal morphology of captive-reared mice; captive-reared mice (two generations bred) had lighter combined kidney and spleen masses compared with wild-caught mice. Sexual dimorphism was maintained in captivity. Our findings demonstrate that captive breeding can alter internal morphology. Given that these morphological changes may impact organismal functioning and viability following release, further investigation is warranted. If the morphological change is shown to be maladaptive, these changes would have significant implications for captive-source populations that are used for reintroduction, including reduced survivorship.

## 1. Introduction

Captive breeding programmes assist in the recovery of threatened taxa by providing supplementary populations or individuals for reintroduction. However, following reintroduction, released individuals often have a low probability of survival [1,2]. Causes of reintroduction failure vary, but have been associated with phenotypic changes in the physiology and morphology of captive-bred animals [3,4].

Morphological change in captivity may result from phenotypic plasticity [5,6]. Plastic changes in morphology can occur in response to environmental conditions during development or from lagged effects of the parental generation [7]. Alternatively, morphological differences may occur due to changes in the strength and targets of selection in captivity, selecting morphological phenotypes that maximize fitness in captivity [6,8,9]. Further, shifts in morphology away from the wild phenotype can occur with each subsequent generation maintained in captivity [3]. For example, oldfield mice (*Peromyscus polionotus subgriseus*) maintained in captivity for differing periods (2, 14 and 35 generations) showed an increased magnitude of change in cranial and mandibular size and shape with each subsequent captive generation [9].

Changes to sexual selection may also occur in captivity and there is a growing interest in how this may influence morphology [3,10]. Sexual dimorphism typically results from morphological traits being favoured by either intra- or inter-sexual selection (e.g. body size) [11,12]. Reduced competition for resources and artificial selection for animals suited to captivity may inadvertently lead to morphological change; in turn, this may impact morphology with changes to or a reduction in sexual dimorphism [3,12]. Body size is one morphological trait known to change in captivity. For example, captive American mink (*Mustela vison*) had reduced sexual dimorphism in body size and craniometric variation, likely due to relaxed sexual selection [13]. However, empirical evidence of morphological change for each sex in captivity is largely limited to a small number of studies in birds and fish [3].

In captivity, animals face changes in various environmental conditions, but the most pronounced are associated with diet and nutrition [3], social interactions [14] and degree of cognitive stimulation [15]. Changes in such factors are known to lead to changes in external morphological traits [14,16,17] and skeletal traits [9,10,18]. However, captivity can also drive changes in soft tissue morphology [9], with empirical studies beginning to document changes in the size and shape of the brain [19,20] and the digestive tract [3,14,21].

Critically, the extent of external and internal morphological changes may differ in both direction and magnitude [3]. Specifically, subtle external changes may mask more pronounced internal changes. For example, when compared with wild mallards (*Anas platyrhynchos*), captive-reared mallards showed no significant differences in external morphology, but the captive birds had lighter gizzard mass [21]. These internal changes impacted the captive-reared mallards following release; they were unable to reach a necessary body condition following release, which reduced their probability of survival [21]. Given the potentially fatal consequences of undetectable changes in internal morphology, studies attempting to investigate the influence of captivity on morphology should aim to quantify changes in both external and internal morphology. Such research should provide important insights into the types of morphological traits most susceptible to change [9], and more importantly, whether such plasticity could be used to generate animals in captivity that might have higher chances of survival post-release [4].

Changes in internal morphology are of interest because they are the major interface between an organism and the environment [22]. Further, internal changes can have major impacts on organismal functioning and viability. For example, captive animals have little to no exposure to parasites, requiring reduced immune responses in captivity [23–25]. Therefore, organs such as the spleen and small intestine known to elicit changes in response to parasitism may reduce in size to maximize their functional capacity in the captive environment [26,27]. In addition, food provided in captivity is likely to be higher in nutrient and energy density and more freely available compared with natural conditions [28–30]. The changes in resource availability and quality may change the demands placed on an animal's gastrointestinal tract, thus eliciting changes in the small intestine, as well as the kidneys and spleen [22,31–36].

While future studies would benefit from focusing on key morphological traits critical for post-release fitness, we firstly need to identify what morphological traits might change in captivity. Future studies can then explicitly compare or manipulate environmental factors in captivity to provide robust inferences about the mechanisms for morphological change in captivity. The broad aim of this study was to provide a holistic assessment of the effects of captivity on the internal and external morphology of house mouse (*Mus musculus*) as a model species for small mammals. The specific aims were: (i) to compare the external and internal morphological traits between captive-reared and wild-caught individuals; (ii) to examine the effect of captivity on external and internal morphology across captive generations; and (iii) to compare the internal and external morphology of each sex from the captive and wild environments.

## 2. Material and methods

### 2.1. Study species

The house mouse is a small rodent distributed globally; the wild-derived strain was used in this study. This species is easily maintained in captivity and has a short generation time, which permits multiple generation studies to be conducted over relatively short periods [3]. Further, *M. musculus* provides a good model for investigating the effects of captivity on small mammals, because it shares a number of life-history traits in common with other small mammals. These include short generation time, high reproductive value, large litter sizes, iteroparity, sexual dimorphism and early age at maturity [37–42]. For these reasons, *M. musculus* is being increasingly used as a model to address questions related to small mammal captive breeding and reintroduction [3,43].

### 2.2. Housing and feeding

All individuals (wild-caught and captive-reared) were maintained separately in opaque plastic cages with a metal top (32 × 18 × 12 cm; MB1 Mouse Box, Wiretainers Pty Ltd, Melbourne, Victoria, Australia). We used wood shavings as cage substrate and all cages were provided with bedding material (shredded paper) and a 6 × 4 cm cardboard tube for cover. Water and food (Vella Stock Feeds Brand Rat and Mouse Nut; The Vella Group, Glendenning, New South Wales, Australia) were available *ad libitum*, determined as 20 g of food per 100 g of body mass supplied daily [44]. Room temperature was maintained at 22 ± 2°C on a 12 hour reverse light : dark cycle, lights on at 9:00am AEST, with full spectrum UV light provided. Humidity was not controlled; however it was monitored daily and recorded as 75 ± 10%. Animals were monitored daily, with cages cleaned once a week by removing the occupant and placing it in a round escape-proof container (54 × 52 cm; Spacepac Industries Pty Ltd, Wollongong, NSW, Australia) before placement in a new cage.

### 2.3. Captive-reared F_4_ generation

Eleven virgin adult male and fifteen virgin adult female *M. musculus* were sourced from an existing captive population maintained at the University of New South Wales (UNSW), Sydney, under Ethics Permit UNSW Reg. No. 12/88A. All mice were third or fourth generation captive-reared mice born between late 2012 and mid-2013. No individuals shared related parents or grandparents descended from the original wild-caught founder generation. The original population consisted of 42 females and 45 males captured between March and May 2011 at an agricultural site in the western Sydney area, NSW, Australia (34°4′36.48″ S, 150°34′15.6″ E), where March–May temperatures averaged 24.0–26.3°C. Prior to relocation to the University of Wollongong, captive-reared F_4_ mice were housed at UNSW in a temperature (19–25°C) and light-controlled room (12 : 12 h reverse light cycle, lights on at 9:00am AEST). Humidity was not controlled but was approximately 30% (A. Gibson 2014, personal communication). Males were housed separately at weaning but female siblings were housed together in groups of up to three individuals. All animals had been provided with food and water *ad libitum*. Mice were monitored daily and thoroughly checked three times a week for body condition, injuries and behaviour.

For this study, captive-reared F_4_ individuals were collected late January 2014 and transported to the Ecological Research Centre at the University of Wollongong, Wollongong, NSW. Mice were weighed (grams) on digital scales (Mettler-Toledo PJ3600, Mettler-Toledo Ltd, Port Melbourne, Victoria, Australia) upon entry into the individual housing (see Housing and feeding).

Once acclimated to the individual housing, captive-reared F_4_ individuals were used to breed the F_5_ generation. At the conclusion of the breeding period, captive-reared F_4_ individuals were then re-acclimated to the individual housing for a minimum period of 12 days before quantifying external and internal morphological traits (see External and internal morphological traits).

### 2.4. Captive-reared F_5_ generation

Pedigree mapping was used to ensure that individuals from the founder generation were paired so that captive-reared F_5_ mice did not share related parents or grandparents. Monogamous breeding pairs were held together for one week. Each breeding pair was housed in the same caging used for all wild-caught and captive-reared individuals in this study (see Housing and feeding).

Once mated, the captive-reared F_4_ mothers were minimally disturbed, but were closely monitored on a daily basis around the expected due date to check for young. Offspring were housed with their mother until they were weaned at 25 days of age; this was kept uniform across all litters to reduce differences in maternal investment post-pregnancy. At 25 days of age, the captive-reared F_4_ mother was removed from the breeding cage, and the litter housed for 2 days under *ad libitum* conditions; this was done with the view to reduce post-weaning stress on the litter. After 2 days, the offspring were then housed individually in the same caging used for all wild-caught and captive-reared individuals in this study (see Housing and feeding). The sex of each offspring (henceforth, captive-reared F_5_) was determined as the mouse was placed in its individual housing (13 males and 14 females). Captive-reared F_5_ mice were individually housed until they reached sexual maturity before quantifying external and internal morphological traits (see External and internal morphological traits).

### 2.5. Wild-caught population

Eight adult male and fifteen adult female *M. musculus* were captured in October–November 2014, at the same agricultural site in the western Sydney area (34°4′36.48″ S, 150°34′15.6″ E) as the source population of the original wild-caught founder generation (see Captive-reared F_4_ generation). Elliott traps (30 × 10 × 8 cm; Sherman Traps Inc., Florida, USA) were set inside and outside sheds and surrounding vegetation. These were checked and emptied daily in the early morning approximately 8.00 am AEST. Elliott traps were baited with honey and peanut butter rolled oat balls.

Once captured, animals were transported to the Ecological Research Centre at the University of Wollongong, Wollongong (34°24′24″ S 150°52′46″ E) and housed in the same caging as the captive-reared generations (see Housing and feeding). Wild-caught individuals were acclimated to the individual housing for a minimum period of 12 days before quantifying external and internal morphological traits (see External and internal morphological traits).

### 2.6. External and internal morphological traits

Animals were euthanased using CO_2_ asphyxiation. Immediately following euthanasia, external morphological trait measurement and macroscopic dissection of organs were conducted to study morphometric differences between wild-caught and captive-reared F_4_ and F_5_ generations. External traits were: body mass (grams), skull length, body length, tail length and foot length (right hind leg; millimetres). Internal traits were: mass of brain, liver, combined kidneys, heart, lungs, testes/ovaries, spleen, stomach, caecum, small and large intestine and the lengths of the small and large intestine. Organs were weighed using scales with ± 0.01 g precision (Mettler-Toledo PJ3600, Mettler-Toledo Ltd, Port Melbourne, Victoria, Australia). Where applicable, digestive organs were emptied of their contents and rinsed with a 0.9% saline solution and weighed. The lengths of the small and large intestine were measured using slide callipers with ± 0.05 mm precision.

## 3. Statistical analysis

### 3.1. Multivariate analysis

To examine the effects of rearing environment on the external and internal morphology of mice, we used permutational analysis of variance (PERMANOVA) with 9999 permutations in Primer 7 (PRIMER-E Ltd, Plymouth, UK) [45] and PERMANOVA+ B version [46]. Permutational analyses were selected in favour of parametric analyses for these data because they are suitable for small and unequal sample sizes when comparing treatments [47–49] and for examining changes in morphology across multiple generations [50].

To control for the effects of body size on morphological traits, we calculated the residuals of a least-squares regression of each morphological trait on body size using body mass or body length where lengths were measured. We then normalized the morphological trait data so that all morphological traits would take values within the same limits (−2 to +2 to cover all entries).

To test whether morphological traits differed between rearing environment and sex, a two-factor PERMANOVA was used on the external and internal morphological traits. In this analysis, the factors were rearing environment (three levels orthogonal and fixed; wild-caught; captive-reared F_4_ and captive-reared F_5_) and sex (two levels orthogonal and fixed; female and male), with acclimation period (number of days acclimated) included as a covariate. An interaction term between rearing environment and sex was also included to account for any interactive effects of rearing environment and sex on morphology. All analyses used Euclidean similarity measures. Where significant in the main PERMANOVA test, PERMANOVA pairwise comparisons were conducted to compare external and internal morphology between rearing environments and between males and females. Pairwise comparisons were conducted in PERMANOVA+ B version [46]. Similarity percentage (SIMPER) analysis was used to identify the morphological traits that were primarily responsible for the compositional differences in external and internal morphology between captive-reared F_5_, captive-reared F_4_ and wild-caught animals and between males and females. Only traits that contributed greater than 10% to compositional changes were used in univariate analyses, as these traits were likely to be primarily responsible for the compositional differences. One individual was excluded from external and internal morphological trait SIMPER analysis due to missing morphometric values.

### 3.2. Univariate analyses

To examine the effects of sex on external morphology of mice, four external morphological traits that contributed greater than 10% to compositional changes in external morphology between sexes in SIMPER were analysed using analysis of variance (ANOVA, table 3; electronic supplementary material). To correct *p*-values for multiple testing on external morphological traits, a Bonferroni adjusted alpha level (*α* = 0.0125) was used. To control for the effects of body size on external morphological traits, we used the residuals of a least-squares regression of each morphological trait in analyses. Two individuals were unable to be sampled and subsequently removed from the analysis (one captive-reared F_4_ male due to an unexpected death; one captive-reared F_4_ female with a previously damaged tail). Residuals from ANOVAs were inspected to verify normality and homogeneity of variances. For all morphological data, Tukey's HSD pairwise comparison tests were used for *post hoc* comparisons between treatments. Where normality was unable to be met, Kruskal–Wallis tests were used, with *post hoc* comparisons made using Wilcoxon tests.

To examine the effects of rearing environment and sex on the internal morphology in mice, internal morphological traits that contributed greater than 10% to compositional changes in internal morphology between rearing environments and sex in SIMPER were analysed using analysis of covariance (ANCOVA, table 3; electronic supplementary material). To correct *p*-values for multiple testing on internal morphological traits, a Bonferroni adjusted alpha level was used (*α *= 0.00625). For internal morphology, the effects of rearing environment and sex were the fixed effects, and acclimation period (number of days acclimated) was the covariate. An interaction term between rearing environment and sex was also included. To control for the effects of body size on internal morphological traits, we calculated the residuals of a least-squares regression of each morphological trait on body mass (or body length where length was measured). Where individuals were unable to be sampled for specific internal morphological traits, the degrees of freedom for these respective analyses were adjusted to account for these exclusions. Residuals from ANCOVAs were visually inspected to verify normality and homogeneity of variances. As there was no interaction between rearing environment and sex on any internal morphological traits, ANOVAs were then conducted to estimate the effect of rearing environment or sex on internal morphological traits (brain, liver, combined kidneys, spleen, small intestine length, large intestine, large intestine length, caecum) showing significance in the ANCOVA. Where the assumptions of normality and/or homogeneity of variance were not met, Kruskal–Wallis tests were used, with *post hoc* comparisons made using Wilcoxon tests. All morphological data were analysed in the JMP 11.2.0 statistical package.

## 4. Results

### 4.1. Effects of rearing environment and sex on morphology

There was a significant interaction between rearing environment and sex on internal morphology (Internal: Pseudo-F: 1.926, *p* = 0.018; table 1). There was no significant interaction between rearing environment and sex on external morphology (External: Pseudo-F: 1.997, *p* = 0.081; table 1). Further, there were no significant interactions between acclimation period, rearing environment and/or sex on external or internal morphology (table 1).

**Table 1.** PERMANOVA analyses testing the effects of rearing environment (rearing env.), sex and acclimation period (accl.) on external and internal morphology. Significant *p*-values indicated by asterisk.

	external				internal		
	d.f.	MS	Pseudo-F	P(perm)	MS	Pseudo-F	P(perm)
Accl. × Rearing env. × Sex	2	2.582	0.543	0.737	8.991	0.895	0.558
Accl. × Rearing env.	2	2.062	0.434	0.848	12.516	1.246	0.226
Accl. × Sex	1	8.168	1.717	0.153	17.243	1.717	0.080
Accl.	1	3.766	0.792	0.528	87.151	8.678	<0.0001*
Rearing env. × Sex	2	9.499	1.997	0.081	19.343	1.926	0.018*
Rearing env.	2	7.000	1.472	0.135	28.648	2.853	0.004*
Sex	1	16.176	3.401	0.009*	63.232	6.296	<0.0001*
Residual	64	4.756			10.043		

*p < 0.05.

External morphology did not significantly differ between rearing environments and acclimation period (External–Rearing environment: Pseudo-F = 1.472, *p* = 0.135; Acclimation period: Pseudo-F = 0.792, *p* = 0.528; table 1). However, external morphology did significantly differ between male and female mice (External–Sex: Pseudo-F = 3.401, *p* = 0.009; table 1).

The internal morphology significantly differed between individuals from differing rearing environments (Internal–Rearing environment: Pseudo-F = 2.853, *p* = 0.004; table 1), between sex (Internal–Sex: Pseudo-F = 6.296, *p* < 0.0001; table 1) and acclimation period (Internal–Acclimation period: Pseudo-F = 8.678, *p* < 0.0001; table 1). SIMPER analysis revealed four external and nine internal morphological traits were driving the compositional differences in external and internal morphology between captive-reared F_4_, captive-reared F_5_ and wild-caught individuals and sex (only morphological traits with greater than 10% contribution were considered; table 3; electronic supplementary material).

### 4.2. Effect of captivity on internal morphology across multiple generations

The sizes of the internal organs significantly differed between captive-reared F_5_ and captive-reared F_4_ females (*t*_25 _= 1.650, *p* = 0.007; table 2) and between captive-reared F_5_ and wild-caught females (*t*_25 _= 1.805, *p* = 0.001; table 2). There was no significant difference between captive-reared F_4_ and wild-caught females (*t*_26 _= 1.094, *p* = 0.293; table 2). SIMPER analysis revealed five morphological traits (brain, combined kidney mass, stomach, caecum and ovaries; electronic supplementary material) were driving compositional differences in internal morphology between captive-reared F_5_ and captive-reared F_4_ females.

**Table 2.** PERMANOVA pairwise tests comparing external and internal morphology between rearing environments and sex. Significant *p*-values indicated by asterisk.

pairwise tests	*t*	Den. d.f.	P (perm)
external morphology			
female, male	1.844	64	0.009*
internal morphology			
F_5_ female, F_4_ female	1.650	25	0.007*
F_5_ female, wild female	1.805	25	0.001*
F_4_ female, wild female	1.094	26	0.293
F_5_ male, F_4_ male	1.186	20	0.219
F_5_ male, wild male	1.151	17	0.223
F_4_ male, wild male	0.996	15	0.434
F_4_ female, F_4_ male	2.026	22	0.002*
F_5_ female, F_5_ male	1.674	23	0.004*
wild female, wild male	1.588	19	0.012*

*p < 0.05.

The sizes of the internal organs did not significantly differ between captive-reared F_5_ and captive-reared F_4_ males (*t*_20 _= 1.186, *p* = 0.219; table 2) or between captive-reared F_5_ and wild-caught males (*t*_17 _= 1.151, *p* = 0.223; table 2). Further, there was no significant difference between captive-reared F_4_ and wild-caught males (*t*_15 _= 0.996, *p* = 0.434; table 2). SIMPER analysis revealed four morphological traits (large intestine, large intestine length, lungs and caecum; electronic supplementary material) were driving compositional differences in internal morphology between captive-reared F_5_ and captive-reared F_4_ males.

### 4.3. Sexual dimorphism in external and internal morphology

The external morphology differed significantly between female and males (*t*_64 _= 1.884, *p* = 0.009; table 2), SIMPER analysis revealed body mass, body length, skull and tail lengths were driving compositional differences in external morphology between the sexes (electronic supplementary material). Only body mass differed significantly between females and males in external morphological traits (*χ*^2^_1 _= 10.296, *p *= 0.001; table 3).

**Table 3.** Effect of rearing environment and sex on external and internal morphological traits in house mouse. Statistical output from ANOVA for external morphological traits, output from ANCOVA for internal morphological traits. Bonferroni corrected probabilities are shown with asterisks. *P*-values include adjusted *α* levels.

	Rearing Environment × Sex			Rearing environment			Sex				Acclimation period		
	*F*	d.f.	*p*	*F*	d.f.	*p*	*χ*^2^	*F*	d.f.	*p*	*F*	d.f.	*p*
external morphological traits													
body mass (g)							10.296		1	0.001^a^			
body length (mm)								3.331	1, 74	0.072			
foot length (mm)								0.289	1, 74	0.592			
tail length (mm)							0.772		1	0.380			

	Rearing Environment × Sex			Rearing environment			Sex				Acclimation period		
	*F*	d.f.	*p*	*F*	d.f.	*p*	*χ*^2^	*F*	d.f.	*p*	*F*	d.f.	*p*
internal morphological traits													
brain (g)	0.324	2, 68	0.724	0.655	2, 68	0.523		11.229	1, 68	0.001^b^	0.441	1, 68	0.509
liver (g)	1.262	2, 68	0.289	2.624	2, 68	0.079		4.033	1, 68	0.047	9.899	1, 68	0.003^b^
combined kidneys (g)	2.346	2, 68	0.104	6.711	2, 68	0.002^b^		47.262	1, 68	<0.0001^b^	1.332	1, 68	0.253
spleen (g)	2.426	2, 68	0.096	5.433	2, 68	0.006^b^		0.111	1, 68	0.740	4.005	1, 68	0.049
small intestine length (mm)	0.286	2, 68	0.752	4.670	2, 68	0.012		0.611	1, 68	0.437	0.809	1, 68	0.372
large intestine (g)	2.592	2, 68	0.082	3.138	2, 68	0.049		0.708	1, 68	0.403	1.269	1, 68	0.264
large intestine length (mm)	2.713	2, 68	0.074	2.408	2, 68	0.098		8.644	1, 68	0.004^b^	1.554	1, 68	0.217
caecum (g)	0.384	2, 68	0.683	5.355	2, 68	0.007		0.042	1, 68	0.839	0.016	1, 68	0.900

^a^External morphological traits significant after a Bonferroni adjusted *α* level (*α *= 0.0125).

^b^Internal morphological traits significant after a Bonferroni adjusted *α* level (*α *= 0.00625).

The internal morphology of captive-reared F_4_ females and males differed significantly (*t*_22 _= 2.026, *p* = 0.002; table 2), and SIMPER analysis revealed that large intestine length, combined kidney and large intestine masses were driving this compositional difference. Furthermore, captive-reared F_5_ female and males differed significantly in their internal morphologies (*t*_23 _= 1.674, *p* = 0.004; table 2), with caecum, brain and stomach masses driving compositional differences in internal morphology between captive-reared F_5_ females and males. Wild-caught female and males differed significantly (*t*_19_ = 1.588, *p*= 0.012; table 2); SIMPER analysis revealed that liver, spleen and combined kidney masses were driving compositional differences in internal morphology between wild-caught females and males (electronic supplementary material).

### 4.4. Effects of rearing environment and sex on morphological traits

There was no significant interaction between rearing environment and sex on any internal morphological traits (table 3). Acclimation period had a significant effect on liver mass (*F*_1, 68 _= 9.899, *p* = 0.003; table 3).

Three internal morphological traits and one external morphological trait contributing greater than 10% to compositional differences in external and internal morphology were significant between females and males following Bonferroni adjustment in the ANCOVA (table 3; electronic supplementary material). The body mass (*χ*^2^_1 _= 10.296, *p*= 0.001), brain (*F*_1, 68 _= 11.229, *p *= 0.001) combined kidney masses (*F*_1, 68 _= 47.262, *p* < 0.0001) were significantly lighter and large intestine length (*F*_1, 68 _= 8.644, *p* = 0.004) significantly shorter in females compared with male house mouse (tables 3 and 4).

**Table 4.** Effect of sex on external and internal morphological traits in house mouse. Statistical output from ANOVA for morphological traits. Values are raw values mean ± s.e. Different superscript letters denote statistically significant differences across rows. Bonferroni corrected probabilities are shown as footnote. *P*-values include an adjusted *α* level (*α *= 0.0125).

	female (*n* = 44)	male (*n* = 32)	*F*	*χ*^2^	d.f.	*p*
body mass (g)	13.164 ± 0.380^A^	15.788 ± 0.667^B^		10.296	1	0.001^a^
brain (g)	0.381 ± 0.005^A^	0.383 ± 0.007^B^	14.039		1	0.0004^a^
combined kidneys (g)	0.231 ± 0.007^A^	0.334 ± 0.015^B^	41.973		1	<0.0001^a^
large intestine length (mm)	80.568 ± 1.371^A^	88.226 ± 1.773^B^	7.869		1	0.006^a^

^a^Significant under a Bonferroni adjusted *α* level (*α *= 0.0125).

There was a significant effect of rearing environment on combined kidney and spleen mass (table 3). The combined kidney and spleen mass were lighter in captive-reared F_5_ compared with captive-reared F_4_ individuals (Combined kidney: *F*_2, 68 _= 6.711, *p* = 0.002; Spleen: *F*_2, 68 _= 5.433, *p* = 0.006; table 5). Further, there was evidence that multiple generations maintained in captivity results in a shift away from wild phenotypes; combined kidney mass was lighter in captive-reared F_5_ compared with wild-caught individuals (table 5).

**Table 5.** Effect of rearing environment on internal morphological traits in house mouse. Statistical output from ANOVA for internal morphological traits. Values are raw values mean ± s.e. Different superscript letters denote statistically significant differences across rows. Bonferroni corrected probabilities are shown as footnote. *P*-values include an adjusted *α* level (*α *= 0.00625).

	wild (*n* = 23)	captive F_4_ (*n *= 26)	captive F_5_ (*n* = 27)	*χ*^2^	d.f.	*p*
combined kidneys (g)	0.291 ± 0.020^A^	0.296 ± 0.017^A^	0.239 ± 0.009^B^	10.862	2	0.004^a^
spleen (g)	0.043 ± 0.006^A^	0.019 ± 0.002^B^	0.018 ± 0.001^C^	24.370	2	<0.0001^a^

^a^Morphological traits significant under a Bonferroni adjusted *α* level (*α *= 0.00625).

## 5. Discussion

### 5.1. Effects of captivity on morphology

Captive-reared mice showed differences in internal morphology, but not external morphology, when compared with their wild-caught conspecifics. Differences in morphology between captive and wild environments can be expected due to these environments differing in a multitude of biotic and abiotic factors [19]. The absence of significant changes to external morphology can be explained in one of two possible ways. First, differences between captive and natural environments may induce changes in life-history organization, such as early sexual maturity, instead of changes to somatic growth in external morphological traits. Indeed, this has been observed in hatchery chinook salmon (*Oncorhynchus tshawytscha*), with egg size decreasing across a 20-year period but with no change in female body mass [51]. Second, external morphological traits may be less plastic, with changes in external morphology occurring more slowly and taking multiple generations to manifest [3,9]. Indeed, in captive black-footed ferrets (*Mustela nigripes*), skull and dental traits were 5–6% smaller than wild populations (founder population; museum specimens) and 3–10% smaller than wild-caught populations (collected near the founding population), but these differences only became apparent after more than 10 years of captive breeding [18]. In this case, the captive-reared house mouse used in our study may not have been sufficiently removed from the wild-caught founders (individuals were three to five generations removed) for changes in external morphology to become apparent [9].

In this study, the absence of changes in external morphology masked internal morphological changes. Specifically, combined kidney and spleen mass were lighter in captive-reared individuals compared with wild-caught individuals. The finding that some but not all internal morphological traits changed in captivity (i.e. some traits displayed remarkable consistency between wild and captive populations) may have occurred because not all traits strongly impact individual fitness in the captive environment [9]. This could relax selection pressure for trait change. Alternatively, widespread changes in morphology may require multiple generations to manifest because individual traits differ in how quickly they respond to change. To test this, and demonstrate both the speed and directionality of change, and that changes are biologically relevant, it would be necessary to track changes over additional generations.

Changes in organ sizes occurring in captivity could be due to the functional capacity of these organs being in excess of the actual demands, which would make the organs expensive and inefficient to maintain. Subsequently, the size of organs may have altered to deal with such inefficiency [22,29,52]. For example, changes in the mass or length of the digestive organs may have occurred due to an increased digestive efficiency associated with a higher quality captive diet [21,53], and changes in accessory organs such as kidneys and spleen may have occurred due to decreased immunological and disease exposure in the captive environment [54–57]. However, identifying the specific mechanisms that cause morphological changes can be challenging. This is largely because multiple environmental factors can affect internal morphology, and the effects of these factors are likely to be interactive [28]. Future studies would benefit from explicitly comparing the nutrient and energy content of diets, or even by manipulating these in captivity to provide robust inferences about the mechanisms for morphological change in captivity.

### 5.2. Effect of captivity on morphology across multiple generations

Captive-reared F_5_ individuals had significantly lighter combined kidney and spleen mass compared with captive-reared F_4_ and wild-caught individuals. This may indicate a directional shift away from the wild-caught phenotype across one generation in captivity, but only in internal morphological traits [9]. Previous studies report change in internal morphological traits can occur after just one generation maintained in captivity [14,19]. The lack of significant differences in other internal and external morphological traits between captive-reared F_5_ and captive-reared F_4_ house mouse may indicate that these traits do not play a significant role in fitness in captivity [3,9].

Interestingly, the overall sizes of the internal organs of captive-reared F_5_ male mouse did not differ significantly from captive-reared F_4_ or from the wild-caught males. The reason for this finding is not clear, but sex-based differences in the magnitude of change in response to captivity across generations may explain why male internal morphology was more stable [3,9]. A previous study investigating the effects of selective breeding for high activity in house mouse reported females and males having differing rates of morphological change in response to high activity, indicating that trait plasticity differed between the sexes across generations [55]. Given these findings, changes in internal morphology may take multiple generations to manifest in males [3,9]. If directional changes in internal morphology are shown to occur across multiple generations of mouse maintained in captivity, this is likely to have significant implications for captive-bred animals following release [3]. For example, one study found 83% of offspring post-release were of same-source parentage in released third-generation captive-bred and wild-caught *M. musculus*, suggesting that morphological traits changing in captivity (such as body size) may be important to mate selection and may infer assortative mating [43]. In this case, the captive-bred reintroduced animals appear to have higher reproductive success, but exactly how captivity affects the chances of survival post-release remains to be studied.

### 5.3. Effect of captivity on sexual dimorphism in morphology

Both external and internal morphology were found to differ significantly between females and males, and these sex-based morphological differences occurred in both captive-reared and wild-caught house mouse. While we can expect sex-based differences in morphology as an outcome of sexual selection favouring different trait values in males and females, we expected a loss of sexual dimorphism in captivity due to changes in resource availability and the strengths and targets of sexual selection [3,13]. The maintenance of sexual dimorphism in the present study suggests that sexual selection pressures remained unchanged in the captive environment. Alternatively, changes to or loss of sexual dimorphism may take multiple generations to manifest, and may not have been observed in our study [3,9]. There is emerging evidence that sexual dimorphism can be maintained in captivity; however, most studies have not investigated whether relaxation or reduction in sexual selective pressures occurs in captivity [3,12]. As such, to allow for a greater understanding of the effects of captivity on sexual dimorphism, it would be valuable to test for sex-specific differences in various morphological traits across a diversity of taxonomic groups. In recognition of this possibility, several recent studies have begun to explore whether sexual selection theory can be used to inform management strategies [43,58].

### 5.4. Implications for captive breeding programmes and management

Our finding that negligible changes in external morphology masked significant changes to internal morphology has implications for captive breeding programmes. Changes to internal morphology in captivity are known to impact digestive efficiency [21,53] as well as immune responses and disease resistance [54–57]. Consequently, rapid changes in internal morphology could have severe and unforeseen effects on the viability of small mammals held in captivity. However, this is dependent on what morphological traits change, and whether those changes are maladaptive for natural environments. If the morphological change is shown to be maladaptive, these changes would have significant implications for captive-source populations that are used for reintroduction. While there is currently no information on the effect of internal changes on the post-release viability of small mammals, there is some evidence for these effects in birds [21]. Future research on small mammals would benefit from investigating the extent to which internal morphological changes occurring in captivity are maladaptive under natural conditions, and whether these impacts can be mitigated by manipulating the captive environment.

Importantly, it is unknown whether the observed changes in morphology were due to phenotypic plasticity or changes in selection pressures experienced in captivity. Further, case studies investigating morphological plasticity in captivity are limited [3]. Plastic responses in morphology to changes in environmental conditions are common, and these plastic responses can be fast, repeatable and reversible [17,52,59–61]. If morphological traits are shown to be plastic, this presents an opportunity for strategic management of morphological phenotypes [4]. That is, the captive phenotype may be altered to better suit the wild environment; but tailoring methods (such as pre-release exposure) may be required to increase likelihood of survival following release [62]. For example, post-release survival of pheasants (*Phasianus colchicus*) was higher in those that had been exposed to more natural diets prior to release. One of the mechanisms to explain this increased survivorship was the development of gut morphology (changing intestine and caecum lengths) to suit a natural diet [63]. Alternatively, if morphological change is due to selection pressures in captivity, environmental factors that change the strength or direction of selection pressures could be manipulated to drive selection for favourable morphological phenotypes [3,4,64]. In support of this notion, there is emerging evidence that understanding and controlling selection may be able to mitigate the effects of captivity that influence the success of captive animals released into the wild [64].

## 6. Conclusion

This study aimed to investigate whether morphology differed between captive-reared and wild-caught individuals, the effect of captivity on external and internal morphology across generations and whether the sexes responded differently to the captive-rearing environment. The absence of changes to external morphology masked internal morphological changes. Moreover, there was evidence for a shift in internal morphology across one generation in captivity. It was found that morphology significantly differed depending on sex, and that sex-based morphological differences were maintained in the captive-rearing environment. Identifying the magnitude and direction of morphological changes in captivity is an important first step towards developing and refining methodologies to minimize unfavourable phenotypic changes in captivity. In turn, this knowledge may be used to improve captive breeding and reintroduction programmes. Overall, these findings draw attention to the need to consider the potential for both internal and external morphological changes when developing methods to improve captive breeding programmes and reintroduction programmes.

## Supplementary Material

Supplementary data of morphological traits contributed most to similarity in SIMPER analyses

## Data accessibility

Data can be accessed at the Dryad Digital Repository (https://dx.doi.org/10.5061/dryad.p2m0d) [65]. Additional supporting information may be found in the electronic supplementary material.
