# Supplementary material for: Effect of captivity on morphology: negligible changes in external morphology mask significant changes in internal morphology
Source: R Soc Open Sci. 2018 May 9;5(5):172470. doi: 10.1098/rsos.172470 (PMC5990819; doi:10.1098/rsos.172470)
Supplement: Supplementary data of morphological traits contributed most to similarity in SIMPER analyses [file rsos172470supp1.docx]

**Supplementary data**

**Table S1.** Morphological traits that contributed most to similarity in external morphology between sexes (female and male; based on the SIMPER procedure using normalised data of least squares regression for each morphological trait on body mass or body length where lengths were measured).

**Table S2.** Internal morphological traits that contributed most to similarity in internal morphology between rearing environment (captive-reared F_4_, captive-reared F_5_ and wild-caught individuals) and sex (female and male; based on the SIMPER procedure using normalised data of least squares regression for each morphological trait on body mass or body length where lengths were measured).

**Table S1.**

| Female – Average squared distance= 5.45 | | | | | | |  |
| --- | --- | --- | --- | --- | --- | --- | --- |
| Morphological trait | Av. Value | Av. Sq. Dist. | Sq. Dist. /SD | % Contribution | Cumulative % |  | |
| Body mass (g) | -0.309 | 0.555 | 0.48 | 10.17 | 10.17 |  | |
| Skull length (mm) | 0.0873 | 0.88 | 0.48 | 16.13 | 26.3 |  | |
| Body length (mm) | 0.166 | 0.926 | 0.46 | 16.98 | 43.29 |  | |
| Tail length (mm) | -0.0918 | 1.35 | 0.19 | 24.73 | 68.01 |  | |
| Foot length (right hind; mm) | 0.105 | 1.74 | 0.16 | 31.99 | 100 |  | |
|  |  |  |  |  |  |  | |
| Male – Average squared distance= 4.07 | | | | | | | |
| Foot length (right hind) (mm) | -0.132 | 0.0197 | 0.43 | 0.48 | 0.48 |  | |
| Tail length (mm) | 0.123 | 0.533 | 0.36 | 13.08 | 13.56 |  | |
| Body length (mm) | -0.242 | 1.05 | 0.47 | 25.88 | 39.44 |  | |
| Skull length (mm) | -0.0727 | 1.15 | 0.39 | 28.13 | 67.57 |  | |
| Body mass (g) | 0.438 | 1.32 | 0.43 | 32.43 | 100 |  | |
| Female & Male – Average squared distance= 10.12 | | | | | | | |
|  | Female | Male |  |  |  |  | |
|  | Av. Value | Av. Value | Av. Sq. Dist. | Sq. Dist. /SD | % Contribution | Cumulative % | |
| Body mass (g) | -0.309 | 0.438 | 2.38 | 0.64 | 23.5 | 23.5 | |
| Body length (mm) | 0.166 | -0.242 | 2.09 | 0.68 | 20.66 | 44.15 | |
| Skull length (mm) | 0.0873 | -0.0727 | 1.99 | 0.65 | 19.7 | 63.86 | |
| Tail length (mm) | -0.0918 | 0.123 | 1.88 | 0.25 | 18.57 | 82.42 | |

**Table S2.**

| F_4_ Female – Average squared distance = 9.74 | | | | | | |
| --- | --- | --- | --- | --- | --- | --- |
| Morphological trait | Av. Value | Av. Sq. Dist. | Sq. Dist./SD | Contribution % | Cumulative % |  |
| Lungs (g) | -0.28 | 0.285 | 0.52 | 2.93 | 2.93 |  |
| Spleen (g) | -0.411 | 0.366 | 0.49 | 3.76 | 6.69 |  |
| Large intestine (g) | 0.0486 | 0.381 | 0.48 | 3.91 | 10.6 |  |
| Heart (g) | 0.205 | 0.525 | 0.46 | 5.39 | 15.99 |  |
| Liver (g) | -0.00285 | 0.589 | 0.51 | 6.05 | 22.04 |  |
| Combined kidneys (g) | -1.06 | 0.597 | 0.48 | 6.13 | 28.17 |  |
| Small intestine (g) | 0.0747 | 0.671 | 0.49 | 6.9 | 35.06 |  |
| Small intestine length (mm) | -0.746 | 0.786 | 0.51 | 8.08 | 43.14 |  |
| Caecum (g) | 0.671 | 0.817 | 0.52 | 8.39 | 51.53 |  |
| Large intestine length (mm) | -0.405 | 0.939 | 0.52 | 9.64 | 61.17 |  |
| Brain (g) | 0.45 | 1.01 | 0.42 | 10.37 | 71.54 |  |
| F_5_ Female – Average squared distance = 5.43 | | | | | | |
| Spleen (g) | -0.0906 | 0.078 | 0.51 | 1.43 | 1.43 |  |
| Liver (g) | 0.163 | 0.0886 | 0.46 | 1.63 | 3.07 |  |
| Large intestine (g) | -0.0211 | 0.0986 | 0.52 | 1.81 | 4.88 |  |
| Small intestine length (mm) | -0.203 | 0.122 | 0.49 | 2.25 | 7.13 |  |
| Combine kidneys (g) | 0.0955 | 0.14 | 0.53 | 2.58 | 9.72 |  |
| Small intestine (g) | -0.0922 | 0.278 | 0.43 | 5.12 | 14.83 |  |
| Ovaries/testes (g) | -0.0772 | 0.321 | 0.45 | 5.91 | 20.74 |  |
| Lungs (g) | 0.045 | 0.332 | 0.46 | 6.1 | 26.84 |  |
| Large intestine length (mm) | -0.253 | 0.601 | 0.46 | 11.05 | 37.9 |  |
| Caecum (g) | -0.03 | 0.632 | 0.46 | 11.62 | 49.52 |  |
| Stomach (g) | 0.241 | 0.781 | 0.53 | 14.38 | 63.9 |  |
| Heart (g) | 0.0158 | 0.847 | 0.47 | 15.58 | 79.48 |  |
| Wild Female – Average squared distance = 11.85 | | | | | | |
| Caecum (g) | -0.596 | 0.286 | 0.4 | 2.42 | 2.42 |  |
| Ovaries/testes (g) | -0.996 | 0.288 | 0.5 | 2.43 | 4.84 |  |
| Combined kidneys (g) | -0.505 | 0.335 | 0.33 | 2.83 | 7.67 |  |
| Small intestine length (mm) | 0.864 | 0.572 | 0.43 | 4.83 | 12.5 |  |
| Brain (g) | 0.35 | 0.617 | 0.52 | 5.21 | 17.71 |  |
| Heart (g) | 0.188 | 0.669 | 0.51 | 5.65 | 23.36 |  |
| Spleen(g) | 0.623 | 0.934 | 0.33 | 7.88 | 31.24 |  |
| Stomach (g) | -0.13 | 1.09 | 0.49 | 9.24 | 40.48 |  |
| Lungs (g) | 0.383 | 1.1 | 0.48 | 9.25 | 49.73 |  |
| Large intestine length (mm) | -0.123 | 1.19 | 0.44 | 10 | 59.73 |  |
| Small intestine (g) | 0.549 | 1.33 | 0.35 | 11.25 | 70.99 |  |
| F_4_ Male – Average squared distance = 14.66 | | | | | | |
| Spleen (g) | -0.887 | 0.292 | 0.49 | 1.99 | 1.99 |  |
| Ovaries/testes (g) | -0.216 | 0.339 | 0.43 | 2.31 | 4.3 |  |
| Liver (g) | -0.496 | 0.349 | 0.48 | 2.38 | 6.69 |  |
| Small intestine (g) | -1.25 | 0.505 | 0.52 | 3.45 | 10.13 |  |
| Stomach (g) | -0.3 | 0.577 | 0.59 | 3.94 | 14.07 |  |
| Combined kidneys (g) | 0.508 | 0.644 | 0.44 | 4.4 | 18.47 |  |
| Brain (g) | -0.524 | 0.702 | 0.48 | 4.79 | 23.26 |  |
| Small intestine length (mm) | -0.422 | 0.709 | 0.5 | 4.83 | 28.09 |  |
| Large intestine length (mm) | 1.06 | 0.907 | 0.48 | 6.19 | 34.28 |  |
| Caecum (g) | 0.485 | 1.33 | 0.46 | 9.1 | 43.38 |  |
| Heart (g) | -0.354 | 2.4 | 0.4 | 16.38 | 59.77 |  |
| Lungs (g) | -0.131 | 2.79 | 0.38 | 19.06 | 78.83 |  |
| F_5_ Male – Average squared distance = 7.25 | | | | | | |
| Spleen (g) | -0.111 | 0.217 | 0.48 | 2.99 | 2.99 |  |
| Small Intestine length (mm) | -0.263 | 0.223 | 0.44 | 3.08 | 6.07 |  |
| Combined kidneys (g) | 0.758 | 0.373 | 0.54 | 5.15 | 11.22 |  |
| Liver (g) | -0.462 | 0.382 | 0.41 | 5.28 | 16.5 |  |
| Heart (g) | -0.316 | 0.399 | 0.57 | 5.51 | 22.01 |  |
| Lungs (g) | -0.476 | 0.416 | 0.49 | 5.74 | 27.75 |  |
| Small intestine (g) | -0.179 | 0.434 | 0.54 | 5.98 | 33.73 |  |
| Large intestine (g) | -0.257 | 0.521 | 0.53 | 7.19 | 40.92 |  |
| Ovaries/testes (g) | 0.865 | 0.685 | 0.46 | 9.46 | 50.37 |  |
| Stomach (g) | -0.144 | 0.815 | 0.5 | 11.24 | 61.62 |  |
| Brain (g) | -0.649 | 0.833 | 0.51 | 11.5 | 73.11 |  |
| Wild Male – Average squared distance = 19.87 | | | | | | |
| Large intestine length (mm) | 0.293 | 0.362 | 0.49 | 1.82 | 1.82 |  |
| Large intestine (g) | -0.717 | 0.465 | 0.45 | 2.34 | 4.16 |  |
| Ovaries/testes (g) | 0.653 | 0.74 | 0.49 | 3.73 | 7.89 |  |
| Small intestine (g) | 0.846 | 0.864 | 0.52 | 4.35 | 12.24 |  |
| Brain (g) | -0.132 | 0.948 | 0.53 | 4.77 | 17.01 |  |
| Caecum (g) | -0.36 | 1.28 | 0.55 | 6.45 | 23.46 |  |
| Lungs (g) | 0.666 | 1.45 | 0.58 | 7.3 | 30.77 |  |
| Combined kidneys (g) | 0.903 | 1.63 | 0.48 | 8.2 | 38.96 |  |
| Small intestine length (mm) | 1.09 | 1.72 | 0.51 | 8.63 | 47.6 |  |
| Stomach (g) | 0.115 | 1.8 | 0.45 | 9.04 | 56.63 |  |
| Heart (g) | 0.193 | 2.27 | 0.56 | 11.41 | 68.04 |  |
| Liver (g) | 0.399 | 2.89 | 0.5 | 14.55 | 82.59 |  |
| F_4_ Female & F_5_ Female – Average squared distance = 16.69 | | | | | | |
|  | F_4_ Female | F_5_ Female |  |  |  |  |
|  | Av. Value | Av. Value | Av. Sq. Dist. | Sq. Dist./SD | Contribution % | Cumulative % |
| Brain (g) | 0.45 | 0.195 | 2.04 | 0.75 | 12.24 | 12.24 |
| Combined kidneys (g) | -1.06 | 0.0955 | 2.03 | 1 | 12.14 | 24.38 |
| Stomach (g) | 0.168 | 0.241 | 1.95 | 0.72 | 11.65 | 36.03 |
| Caecum (g) | 0.671 | -0.03 | 1.84 | 0.77 | 11.02 | 47.06 |
| Ovaries/testes (g) | 0.115 | -0.0772 | 1.71 | 0.58 | 10.22 | 57.28 |
| Large intestine length (mm) | -0.405 | -0.253 | 1.46 | 0.74 | 8.73 | 66.01 |
| Heart (g) | 0.205 | 0.0158 | 1.31 | 0.77 | 7.86 | 73.86 |
| F_4_ Female & Wild Female - Average squared distance = 27.95 | | | | | | |
|  | F_4_ Female | Wild Female |  |  |  |  |
| Small intestine length (mm) | -0.746 | 0.864 | 3.86 | 0.93 | 13.82 | 13.82 |
| Ovaries/testes (g) | 0.115 | -0.996 | 2.87 | 0.61 | 10.29 | 24.1 |
| Caecum (g) | 0.671 | -0.596 | 2.63 | 0.79 | 9.43 | 33.53 |
| Liver (g) | -0.00285 | 0.369 | 2.54 | 0.6 | 9.07 | 42.6 |
| Stomach (g) | 0.168 | -0.13 | 2.32 | 0.73 | 8.32 | 50.92 |
| Spleen (g) | -0.411 | 0.623 | 2.28 | 0.52 | 8.17 | 59.09 |
| Small intestine (g) | 0.0747 | 0.549 | 2.1 | 0.48 | 7.5 | 66.59 |
| Large intestine length (mm) | -0.405 | -0.123 | 2.06 | 0.71 | 7.38 | 73.97 |
| F_4_ Female & F_4_ Male – Average squared distance = 31.23 | | | | | | |
|  | F_4_ Female | F_4_ Male |  |  |  |  |
| Large intestine length (mm) | -0.405 | 1.06 | 3.84 | 0.96 | 12.3 | 12.3 |
| Combined kidneys (g) | -1.06 | 0.508 | 3.6 | 1.04 | 11.53 | 23.83 |
| Large intestine (g) | 0.0486 | 0.649 | 3.51 | 0.41 | 11.24 | 35.07 |
| Heart (g) | 0.205 | -0.354 | 2.96 | 0.77 | 9.49 | 44.56 |
| Small intestine (g) | 0.0747 | -1.25 | 2.84 | 0.93 | 9.09 | 53.65 |
| Lungs (g) | -0.28 | -0.131 | 2.8 | 0.45 | 8.97 | 62.62 |
| Brain (g) | 0.45 | -0.524 | 2.52 | 0.81 | 8.08 | 70.7 |
| F_5_ Female & F_5_ Male – Average squared distance = 14.81 | | | | | | |
|  | F_5_ Female | F_5_ Male |  |  |  |  |
| Brain (g) | 0.195 | -0.649 | 2.52 | 0.81 | 17.00 | 17 |
| Ovaries/testes (g) | -0.0772 | 0.865 | 1.82 | 1.16 | 12.27 | 29.27 |
| Caecum (g) | -0.03 | -0.206 | 1.64 | 0.66 | 11.08 | 40.35 |
| Stomach (g) | 0.241 | -0.144 | 1.63 | 0.72 | 10.98 | 51.33 |
| Large intestine length (mm) | -0.253 | -0.116 | 1.35 | 0.7 | 9.12 | 60.44 |
| Heart (g) | 0.0158 | -0.316 | 1.27 | 0.65 | 8.54 | 68.99 |
| Lungs (g) | 0.045 | -0.476 | 0.963 | 0.74 | 6.5 | 75.49 |
| F_4_ Male & F_5_ Male – Average squared distance = 25.73 | | | | | | |
|  | F_4_ Male | F_5_ Male |  |  |  |  |
| Large intestine (g) | 0.649 | -0.257 | 4.1 | 0.43 | 15.91 | 15.91 |
| Lungs (g) | -0.131 | -0.476 | 3.02 | 0.44 | 11.72 | 27.64 |
| Large intestine length (mm) | 1.06 | -0.116 | 2.98 | 0.89 | 11.57 | 39.2 |
| Caecum (g) | 0.485 | -0.206 | 2.7 | 0.78 | 10.5 | 49.71 |
| Heart (g) | -0.354 | -0.316 | 2.53 | 0.54 | 9.84 | 59.54 |
| Ovaries/testes (g) | -0.216 | 0.865 | 2.11 | 0.97 | 8.18 | 67.73 |
| Small intestine (g) | -1.25 | -0.179 | 2 | 0.93 | 7.79 | 75.52 |
| Wild Female & Wild Male – Average squared distance = 34.82 | | | | | | |
|  | Wild Female | Wild Male |  |  |  |  |
| Liver (g) | 0.369 | 0.399 | 4.38 | 0.71 | 12.58 | 12.58 |
| Spleen (g) | 0.623 | 1.05 | 4.08 | 0.79 | 11.73 | 24.3 |
| Combined kidneys | -0.505 | 0.903 | 3.72 | 0.75 | 10.69 | 34.99 |
| Ovaries/testes (g) | -0.996 | 0.653 | 3.64 | 0.94 | 10.45 | 45.44 |
| Stomach (g) | -0.13 | 0.115 | 2.65 | 0.69 | 7.62 | 53.06 |
| Heart (g) | 0.188 | 0.193 | 2.61 | 0.91 | 7.49 | 60.55 |
| Large intestine (g) | 0.143 | -0.717 | 2.51 | 0.51 | 7.21 | 67.75 |
| Lungs (g) | 0.383 | 0.666 | 2.37 | 0.84 | 6.82 | 74.57 |
| F_4_ Male & Wild Male - Average squared distance = 47.16 | | | | | | |
|  | F_4_ Male | Wild Male |  |  |  |  |
| Spleen (g) | -0.887 | 1.05 | 7.05 | 0.81 | 14.94 | 14.94 |
| Small intestine (g) | -1.25 | 0.846 | 5.61 | 1.08 | 11.89 | 26.83 |
| Large intestine (g) | 0.649 | -0.717 | 5.07 | 0.46 | 10.75 | 37.58 |
| Heart (g) | -0.354 | 0.193 | 4.44 | 0.75 | 9.42 | 47 |
| Small intestine length (mm) | -0.422 | 1.09 | 4.43 | 0.8 | 9.38 | 56.38 |
| Lungs (g) | -0.131 | 0.666 | 4.42 | 0.8 | 9.37 | 65.75 |
| Liver (g) | -0.496 | 0.399 | 3.64 | 1.07 | 7.73 | 73.48 |
| F_5_ Male & Wild Male – Average squared distance = 31.42 | | | | | | |
|  | F_5_ Male | Wild Male |  |  |  |  |
| Spleen (g) | -0.111 | 1.05 | 4.58 | 0.75 | 14.58 | 14.58 |
| Liver (g) | -0.462 | 0.399 | 3.62 | 1.01 | 11.53 | 26.11 |
| Small intestine length (mm) | -0.263 | 1.09 | 3.54 | 0.79 | 11.26 | 37.38 |
| Lungs (g) | -0.476 | 0.666 | 2.96 | 0.81 | 9.41 | 46.79 |
| Heart (g) | -0.316 | 0.193 | 2.61 | 0.79 | 8.31 | 55.1 |
| Stomach (g) | -0.144 | 0.115 | 2.39 | 0.69 | 7.61 | 62.71 |
| Small intestine (g) | -0.179 | 0.846 | 2.21 | 0.75 | 7.02 | 69.73 |
| Caecum (g) | -0.206 | -0.36 | 2.17 | 0.69 | 6.91 | 76.64 |
